# It hurts inside: a qualitative study investigating social exclusion and bullying among adolescents reporting frequent pain and high use of non-prescription analgesics

**DOI:** 10.1186/s40359-020-00478-2

**Published:** 2020-10-28

**Authors:** Siv Skarstein, Sølvi Helseth, Lisbeth Gravdal Kvarme

**Affiliations:** Faculty of Health and Science, Oslo Metropolitan University, St. Olavs Plass, P.O. Box 4, 0130 Oslo, Norway

## Abstract

**Background:**

Being bullied and socially excluded interferes with the fundamental need for humans to belong to a social group, which is necessary for well-being. This might underpin pain and the use of non-prescription analgesics.

**Aim:**

The aim of the study was to gain insight into how social exclusion and bullying affect well-being in adolescents reporting frequent pain and high use of non-prescription analgesics.

**Methods:**

A phenomenological hermeneutical method based on in-depth interviews was used. Nineteen teenagers, 14–16 years of age voluntarily participated in the study. Those included had frequent pain and used non-prescription analgesics daily or several times a week.

**Results:**

Bullying and social exclusion affects thoughts, feelings and attitudes and thereby the quality of life of the adolescents we interviewed. They described feelings such as sadness, loneliness and depression, and a sense of being an outsider among peers was common. It also appears that bullying has significant potential to spread in a school environment.

**Conclusion:**

The adolescents were self-aware and well-articulated. They conceptualised their experiences of being socially excluded and bullied. They described being socially excluded and bullied by peers as painful and they used non-prescription analgesics to alleviate pain. Teachers and health professionals should be aware of frequent pain and high use of non-prescribed analgesic medication as signs of psychosocial stress caused by social exclusion and bullying. Social exclusion and bullying should be systematically mapped, and targeted interventions implemented.

## Background

Pain among children and adolescents has been identified as a common and increasingly important social problem [1]. Pain is a complex psychosomatic experience that can be related to different kinds of stressors, such as physical activity, sleep problems, poor eating habits, bullying, harassment, schoolwork pressure, and poor treatment by teachers and peers [2]. Pain has a negative impact on adolescents’ social life, for example in the form of their inability to pursue hobbies, disturbed sleep, absence from school, and inability to meet friends [3]. Stress may be conceptualised as a biological predisposition in the form of headaches or stomach aches, and intervening variables such as cognitive appraisal and coping strategies can affect people’s perception of pain, pain sensitivity, pain experience and pain behaviour [2, 4–6]. An increasing number of teenagers state that they experience a combination of frequent pain, stress, anxiety and depression [7]. Anxiety and depression show a strong relation to chronic pain and may contribute to both the development and preservation of pain and, in turn, to the use of analgesics as a necessary tool for achieving pain relief [7, 8]. Adolescence is considered a particularly vulnerable period when a person goes through the transition from childhood into adulthood. At this stage of life, friends become an important addition to the family network [9, 10].

The concept of quality of life broadly encompasses how an individual measure the ‘goodness’ of multiple aspects of their life. These evaluations include one’s emotional reactions to life occurrences, disposition, sense of life fulfilment and satisfaction, including satisfaction with work and personal relationships [11]. In the literature, the term ‘quality of life’ is also often referred to as well-being [12]. One important aspect of this is that a person’s well-being when young can form the foundation for their quality of life in adulthood [13, 14].

Bullying can be direct, either physically or verbally (saying ‘nasty’ things to a person), or it can also be intentionally hostile [15]. It also encompasses indirect aggression characterised by its typically covert nature and use of third parties, involving, for example, gossiping, spreading malicious rumours and social exclusion, such as deliberately not allowing a person into a group [16]. Being bullied has negative long-term health consequences and it is widely recognised as one of the most significant public health problems in adolescence [17]. According to the World Health Organization’s (WHO) collaborative cross-national survey, 9–13% of children aged 11 to 15 years reported being bullied in the past couple of months [17]. In a meta-analysis on the prevalence of bullying and cyberbullying, the authors estimated a mean prevalence of 35% for traditional bullying (both perpetration and victimisation roles) and 15% for cyberbullying involvement [18]. Social exclusion interferes with the fundamental need for humans to belong to social groups, which is required for their well-being and positive emotions [19]. Studies show that being social excluded and/or bullied can modify stress responses or lead to long-term increases in health problems such as sleep disturbances, headaches, pain and fatigue [13]. According to Olweus [20], bullying occurs in a relationship in which there is an imbalance of power or strength. A robust literature documents that victims of bullying often have low self-esteem and feel depressed, anxious and lonely [21]. Research from developmental psychopathology have shown that stressful life events can lead to the onset and maintenance of depression and anxiety, and that for many adolescents, victimisation is a major life stressor [22]. In addition, peers very often dislike those who are victims, particularly during the middle school years [23]. Depressive symptoms have been found to be significantly associated with analgesic consumption among adolescents [24]. Further, statistics have shown that victimisation from bullying is associated with medicine use, even when controlling for the higher prevalence of symptoms among bullied victims [25]. More specifically, being the victim of bullying is associated with an increased use of analgesics, even after controlling for self-reported pain [26].

To our knowledge, the well-being of adolescents reporting frequent pain and high use of non-prescription analgesics has not been studied through their own stories, including their thoughts, feelings and attitudes. Such knowledge might be useful to teachers and health professionals alike when planning interventions to support adolescents reporting frequent pain and high use of non-prescription analgesics.

### Aim

The aim of the study was to gain insight into how social exclusion and bullying affect well-being in adolescents reporting frequent pain and high use of non-prescription analgesics.

## Methods

A phenomenological hermeneutical method based on in-depth interviews was chosen for the study, an approach that provides insight into the experiences of the participants and provides rich data [27]. This phenomenological hermeneutical method for interpreting interview texts is inspired by the theory of interpretation presented by Paul Ricoeur [28] and further described by Lindseth and Norberg [29]. Such qualitative methods have also been described as ‘the method of choice when straight descriptions of phenomena are desired’ [30, p. 339].

### Participants

Nineteen teenagers, four boys and 15 girls, between 14 and 16 years of age voluntarily participated in the study. Those included had frequent pain, in addition to a daily to several times a week (high) intake of non-prescription analgesics for at least four continuous weeks over the last 12 months, this without having any known diagnosis. The interviews were conducted on a one-to-one basis, each lasting approximately one hour, during 2015, at an office at their school or at the researcher’s workplace during working hours. The participants chose the place for the interview. Participants had to be able to read and understand the Norwegian language. Participants were recruited and interviewed until the research group considered that saturation was reached [31].

### Data collection

The head teacher of 20 randomly chosen schools representing urban, suburban and rural districts in Norway were invited by phone to participate. The head teachers of ten of these schools consented to informing the students at their schools. One researcher visited these ten schools and gave a 15 min presentation of the project to all classes in years 9 and 10, comprising a total of 52 classes with an average of 25 students in each. The adolescents were given written information about the project including a study information package. This package included a combined bookmark/ruler and provided brief information about the study and the researchers’ contact information. It also included information for parents. The participants were informed both verbally and in writing that they were free to withdraw from the project at any time. Those who wanted to participate contacted the researcher by phone. Written and signed informed consent from both the adolescents and their parents was required before inclusion. Twenty adolescents contacted the researcher themselves and nineteen were included. The person not included did not have signed, informed parental consent. Eligible participants comprised students between 14 and 16 years of age who were able to read and understand the Norwegian language. Exclusion criteria were being medically diagnosed with an illness or injury requiring extended use of non-prescription analgesics within the last year. The adolescents did not receive any compensation for participation.

### The thematic interview guide

A thematic interview guide [27] was developed by the research group and addressed the topics: childhood, upbringing, family life and relationship with key family members, use of non-prescription medicines, pain, sleep, eating habits, activities, school life, peer relations and important life events. As regards the use of medicine and pain, the participants were asked: in what situations, how often, how long, who knew about it, their pain, and who they discussed their use of non-prescription pain medication with. As regards peer relations, experiences of social exclusion and being a victim of bullying were explored in detail. The participants were asked exploratory questions that could provide in-depth information about the bullying, such as: when did it start, what was going on, what do you think about the situation, what do you feel about those that bully others, how the bulling affected you, who has witnessed the bulling, and have you received support to stop the bullying. The last questions in the interview were: ‘Is there anything you feel is important to share with me?’; and ‘If you were me, what kind of questions would you ask the participants in this study?’. The interviewer used the thematic interview guide as a checklist. If a theme from the interview guide was missing, the participant was asked whether he or she had reflected upon this theme. However, as far as possible, without losing the focus, the participants were given an opportunity to steer the conversation so that their stories emerged spontaneously. One researcher (a psychiatric nurse) conducted all the one-to-one interviews, either in a meeting room in the researcher’s workplace or at the adolescents’ school. As far as possible, the participants were able to choose the time and place for the interview. Each interview lasted about an hour. Participants were recruited and interviewed until our research group were confident that no new themes were emerging.

The interviews were audio-recorded and transcribed. The NVivo 10 computer program was used to save and organise the audio files and the transcribed material [32].

### Data analysis

The interviews were analysed within the context of self-understanding that refers to the participants’ sayings, which illustrates how they reflect on and understand their own experiences. This is an understanding of the text based on the results of an initial reading of the transcribed narrative interviews. Further, through a structural analysis, the text was divided into meaningful units, condensed and abstracted to form main themes. These main themes were compared with the participants’ responses for validation. The text was then read again as a whole, a process known as critical reading, and the participants’ self-understanding, meaningful units and main themes were reflected on in relation to the researchers’ pre-understanding and relevant literature. During this process, a comprehensive understanding was formulated that also discloses new possibilities [29].

The criterion for validity is whether consensus may be obtained that an interpretation is reasonably documented and logically coherent [33]. In most instances, the research team, comprising one principal investigator and two additional researchers, reached consensus in their interpretation during group discussions. When there was disagreement, the researchers re-assessed the material and reconsidered their perception by discussing the participants’ self-understanding and the main themes again. Through this process, a broader understanding and deeper insight arose.

Our main themes are illustrated by these four headings:

- It started early in school.
- Friends become bullies.
- Bullying is common.
- It hurts inside.

#### Trustworthiness

Representative quotes from the interviews are used to give a more precise representation of what the adolescents expressed. Each of these quotes are viewed as meaningful units and were extracted from the transcripts and translated into English, and then back into Norwegian.

To ensure methodological coherence between data collection, analysis and theoretical understanding, the researchers met regularly to discuss these issues [34].

#### Consent and ethical considerations

The Norwegian Regional Committees for Medical and Health Research Ethics (REC) approved this study in 2012, under study number: 2012/1460a. Unidentifiable material from the study will be stored at the Faculty of Health at Oslo Metropolitan University in Norway until October 2020.

By signing the consent form, the adolescents agreed to consult the school nurse if the interviewer deemed it necessary. School nurses were informed about the study and were prepared to support the adolescents when needed.

## Results

The sample consisted of 19 adolescents, 15 girls and 4 boys, between 14 and 16 years of age. The adolescents we interviewed described a stressful childhood and upbringing, with different strains that often seemed to go on at the same time, such as persistent and serious conflicts between the parents, and mental and physical illness in close family, sometimes in combination with drug abuse. Several had received help from the child welfare service. Many had moved to a new place of residence and changed schools.

The participants spoke about having frequent pain for many years in various parts of the body, and they had all been using non-prescription analgesics daily or several times a week for some time. Pain hampered their everyday lives, and this reduced their opportunity to participate socially and achieve the success they wanted. Further, the participants talked about a lack of energy. Several said that they strived to manage different kinds of stressors in daily life, such as family conflicts, high expectations regarding their own success at school and in sports, and worries about not being healthy and good enough. Socially, they said they felt they had to strive to try to be accepted and to fit in.

The main themes: It started early in school; friends become bullies; Bullying is common; and it hurts inside, were identified as overarching themes from the structural analysis, and will be described in turn below.

### It started early in school

Several reported that bullying had started when they began school, as one girl said: ‘*I wore glasses in my first year at secondary school; they [school mates] called me “brilleslange” [a Norwegian term used in a degrading way as part of bullying of children who wear glasses] and things like that’* (*Girl-42).* One of the boys expressed his early experiences in school in the following way*: ‘The bullying started in the first or second year of school. My classmates were quite violent… so there was a lot of fighting. It was as if the cool boys chose someone…’* When the interviewer asked: *‘Were they physically violent towards you?’*, the boy responded; ‘Yes, they pushed, kicked and hit me*’ (Boy-27).*

### Friends become bullies

Often, one person started the bullying, but other peers often joined in and a harassment gang was formed. Several explained that friends they had previously trusted had turned against them and became part of the gang. In addition to being bullied, it seemed to be extra painful when someone they had trusted and shared experiences with turned against them, as in the case of this girl: *‘People will often pick on you; things you do and how you dress and how you look and all that. She (an earlier friend) has also logged into my Facebook account and had conversations with my friends pretending she was me. She was a very close friend, so it has made it a little more difficult at the same time’ (Girl-29).*

Some girls talked about how they had been victims of sexual harassment. One girl said it had started with her former boyfriend exposing images and stories from their intimate relationship with their classmates. This kind of harassment of a sexual nature spread around and affected more girls in the school environment, and, in time, there was a group of boys who threatened and gained power over a group of girls. One girl explained how bullying spread; first one boy started harassing her, then several boys, and later, also the girls started bullying her. She said that her former group of friends now turned their backs on her when she approached them in the school yard. She described how she felt growing pain in both her head and stomach. Eventually, she had trouble eating and sleeping. Often, she felt so bad that she had to stay home from school. At home, she received non-prescription pain medication from her mother, and when she went to school, she brought this medication with her in case things became painful.

The adolescents’ descriptions of the psychosocial stress among peers varied from being excluded from social settings with peers to being verbally and/or physically harassed by peers. The following is an example that illustrates this variation: *‘They were very outgoing. I was not allowed to participate in their conversations. They wanted to show me that they had fun together. Especially one girl tried to get along with the others; they were originally friends of mine. They said ugly things. It was not physical violence, but I was hurt by what they said. They were really rude’ (Girl-13).*

### Bullying is common

The interviewer witnessed that it was hard for the participants to talk about being socially excluded and sometimes also bullied. Some cried when they talked about it, some spent some time searching for words and most of the participants also tried to find explanations for why this had happened to them.

Several of the adolescents reported that they, in addition to being directly bullied at school, were also subjected to bullying via the internet, as seen in the following: *‘It's the case that Twitter is used for such bullying – it is tweeting that is indirect, but at the same time, it is not indirect, because you realise it is directed right at you, and there’s one insult after the other’ (Girl-29).*

It appeared that several of the interviewees justified the actions of those who excluded and bullied them, as the following quote indicates: *‘But, that's what young children think and do—they don’t think that much about what they say to others’ (Girl-42).* Trivialising and generalising the bullying might create a meaningful explanation for being victimised and thereby reduce the victim’s feeling of embarrassment.

### It hurts inside

The adolescents’ reactions to being a victim of bullying or social exclusion produced negative emotions. Sadness and depression were mentioned as follow-up conditions to bullying, which reduced their well-being, as exemplified in the following: *‘There has been a lot of digital bullying and so on of me … Which really brought me down very recently. I am very often depressed because of that. It has been very uncomfortable to be at school now’ (Girl-29).*

Several reported that social exclusion and bullying affected their well-being in the form of sleeping problems and loss of appetite. One of the girls expressed this as follows: *‘I have a very bad appetite, I can eat a few bites, but I can’t manage more than that’.* Further, she said: *‘I have had great difficulties getting to sleep the last few months, so I am very tired when I'm at school and after school and stuff like that’ (Girl-29).*

Some explained how they used non-prescription analgesics to cope with stressful social contexts where they had to ‘fit in’: *‘It’s very difficult to adapt to everything. You get tired and stressed out. This is very tiring over the teenage years. I think there are many who feel that way, at least girls. That’s why I don’t think it was a coincidence that exactly I started on painkillers. I think it is very much because I felt that way. Yes… it sounds like I'm talking about drugs here. However, you feel painkillers are very, they are so harmless. In addition, it's prescription-free, it’s so easy to get hold of as well. Moreover, it's a very easy solution to things really’ (Girl-25).*

The experience of being different and feeling outside the peer community produces feelings of sadness, as this girl highlights*: ‘I have always been different. I’ve not really fitted into the girl’s community or boy’s community. So yeah, so it's been … you just have to learn to accept it in a way. It was kind of a time where I felt that I… I have been very sad’ (Girl-28).*

Some of the participants, like this girl who had so many days of absence from school that she was not allowed to take her exams, talked about her growing psychosocial problems: ‘*Because I’m very shy, so that I lock myself inside, when the difficult situations first happen. I've isolated myself once or twice a week, lately…………. Yes, I isolate myself very much’ (Girl-29).*

Being accepted and feeling a sense of belonging among peers is very important during adolescence where identities are settled. Thoughts about what it took to be socially accepted were characterised by insecurity and a feeling of not being good enough, as these girls expressed: *‘What I was thinking about all day was what was good and what was bad and how I had behaved. I was very concerned about this…’ (Girl-18). ‘I was very concerned with what I could do to maybe get along and make friends. I was thinking about what was wrong with me, why I was not part of that group of girls’ (Girl-13).*

Uncertainty about how they should behave in order to be included and accepted was experienced as painful and seemed to cause psychosocial stress, as this quote illustrates: *‘I don’t like people having bad thoughts about me. I get very hurt inside when I feel others don’t like me. Therefore, I try to be nice to the person, and I agree, even if I do not agree inside. I find it incredibly horrible when I feel others dislike me or I feel that someone has a grudge against me and stuff like that. Then I generally don’t like to argue and be in such conflicts no matter who or what it is’ (Girl-44).*

## Discussion

The participants are adolescents with frequent pain and who frequently use non-prescription analgesics. Four emerging main themes from the interviews were entitled ‘It started early in school’*, ‘*Friends become bullies’, ‘Bullying is common’ and ‘It hurts inside’.

It is with great interest that we observe how the participants describe their thoughts, feelings and attitudes regarding their own well-being. Their reflections on social exclusion show how they define these situations as bullying. The young people conceptualise their experiences, they are self-aware and well articulated. They describe being socially excluded and bullied by peers as painful and they use non-prescription painkillers to alleviate pain.

Our results show that bullying and social exclusion influence thoughts, feelings and attitudes, and thereby the well-being of the adolescents we interviewed. The adolescents described emotional experiences and feelings such as sadness, loneliness and depression. A feeling of being an outsider among peers was common.

Children who are socially excluded and bullied have more health problems, poorer school adjustment and poorer emotional adjustment than children who have not been bullied [35]. Further, they have increased risk of emotional disorders in adulthood [9, 36]. Adolescence can for some become a life phase where vulnerability manifests, in which physical, psychological and social aspects with significance for well-being later in life can negatively affect and amplify each other.

Some of the participants described using submission and isolation as strategies to avoid being socially excluded and bullied by peers. Negative self-perception is a risk factor in the development of all forms of peer adversities, and vulnerable children often have difficulty making friends because of their own fear of rejection [37]. When someone is socially excluded and/or bullied from early in life, like several of the participants in this study, this can make them especially sensitive and vulnerable to other people’s responses [38]. The participants described employing a coping strategy, albeit maladaptive, as the formation of beliefs that ‘fitting in’ among peers or complying with perceived social norms would protect them. This was the case even if it constrained the adolescents’ own opportunities for self-discovery and exploration of their own choices or resourcefulness [9, 10, 38].

The saying ‘*I was thinking about what was wrong with me*’ illustrates the negative consequences of social exclusion on thoughts and feelings, which in turn can have a negative impact on adolescents’ self-esteem and well-being [39]. Fear of painful feelings and unpleasant emotions might lead to an avoidance of situations that could provoke these experiences. This can in turn result in a lack of possibilities to gain a broader social re-orientation, including exploration of social experiences, development of skills and knowledge relevant to taking on adult social roles, individuation from family, and establishment of an individual identity, which again can negatively affect the adolescents’ lifelong well-being [9, 10, 40–42].

Those who are bullied during childhood are more likely to report depression, social anxiety, social phobia, low self-esteem, and academic problems later in life. Peer victimisation is associated with the internalisation of symptoms such as low self-esteem that in turn leads victims to exclude themselves from social situations [19, 36, 43]. Being a victim of bullying is also found to be associated with greater perceived stress and an increased risk of recurrent pain [44].

Adolescence is important for establishing learned strategies for coping with stress and strains in life [38, 40]. If an adolescent thinks and feels that symptoms of discomfort are alleviated by taking pain medication, this can lead to a stronger belief in the effect of the medicine [40]. Further, a reduced faith in their own resources to handle psychosocial stress might underpin the need for medication [40]. Such belief in the medication, in combination with a learned attitude to use pain medication when experiencing an unpleasant feeling perceived as pain, as well as easy access to the medicine, can underpin the frequency of intake [45, 46].

When friends become enemies, it creates unpredictability and insecurity in everyday life that is detrimental to well-being. It is possible that these adolescents could benefit from individual support and guidance in order to both build up an increased tolerance for perceived stressors and to handle stressful events. Some of the interviewees have been subjected to sexual harassment. Such harassment needs to be uncovered and addressed since, in addition to being stressful to the individual, it seems to have the potential to spread among peers and in a school environment, representing a serious threat to a good, supportive and effective school environment.

When an adolescent is bullied this might be one visible sign that there is serial bullying and multiple victimization going on. Research have also shown that there might be a familial pattern in bullying [57]. Therefore, it is of high importance to investigate the peer community when an induvial reports bullying. Health professionals such as school nurses and teachers should be aware that frequently pain, depression and psychosocial difficulties might origin from bullying [46, 47]. Research have also shown that safe, supportive, and effective schools will reduce school violence. A safe and effective school framework aligns school safety, student support, and academic achievement across individual, classroom, school, and ideally, community levels. The risk and protective factors for academic, social, and behavioral problems are often intertwined; thus, interventions that target one domain frequently impact other domains [54, 55]. The between student–teacher relationships have impact on both physical and verbal bullying between student [56].”

In general, efforts and resources should be targeted to promote a good psychosocial school environment. There are several programmes and techniques available to build a safe, supportive and effective school environment, which can reduce school violence [47–53]. A safe and effective school framework aligns both student support and academic achievement across individual, class, school, and ideally, community levels [54, 55]. In addition, individual and targeted interventions based on mapping the needs of the individual youths can also form the basis for the choice of measures and professional resources. Collaboration between youths, parents, teachers and health professionals, such as school nurses and psychologists, may in such case be necessary.

### Importance

The fact that young people are able to talk about their thoughts, feelings and attitudes related to bullying as a phenomenon could be used actively in relation to forming a community at school with an established language and a self-awareness aimed at counteracting social exclusion and bullying. Early identification, systematic mapping and targeted interventions including social support for adolescents with frequent pain and regular use of non-prescription analgesics might decrease the risk of chronic pain and reduced well-being. Health professionals such as school nurses and teachers should be aware that adolescents experiencing frequent pain, the use of non-prescription analgesics, depression and psychosocial difficulties might originate from bullying [56, 57]. Psychosocial stress caused by bullying and social exclusion should be assessed and monitored. When one adolescent is bullied, this might be a sign that there is serial bullying and multiple victimisation going on. Research has also shown that there might be a familial pattern in bullying [56, 57]. Therefore, it is of high importance to investigate the peer community when an individual reports bullying. The risk and protective factors relating to academic, social and behavioural problems are often intertwined, and interventions that target one domain might therefore impact the others.

We believe that there remains a need to apply knowledge about social exclusion and bullying among adolescents, and further, to implement well-known strategies and develop targeted interventions that are likely to stop social exclusion and bullying and thereby decrease psychosocial stress. This can prevent social exclusion and bullying, increase well-being and thereby both reduce pain and the intake of non-prescription analgesics.

### Limitations

A potential limitation of the present study is that only four of the 19 participants were boys. It is possible that additional, different viewpoints would have been revealed if more boys had been included in our sample. Another limitation of this study is that the ethical requirement to inform parents about the study might have excluded students who did not wish to fully disclose their non-prescription analgesics use to their parents. The main author, who conducted the interviews, is a specialist in psychiatric nursing. During the interviews, she may have used psychiatric interview techniques which revealed more in-depth information, in comparison to other interview approaches. Also, the fact that the interviewer was female may have made the girls, which comprised most of the interviewees, feel more comfortable and willing to disclose information than the boys. However, in relation to the purpose of this study, we believe that the sample is relevant and provides useful information on the well-being of adolescents who have frequent pain and a high use of non-prescription analgesics. Many of the participants repeated statements that form the basis of the main findings. As researchers, we have emphasised the importance of providing a solid and transparent description of both the participants and the research process, thereby enabling the reader to assess whether our findings are transferable to their setting. We consider it highly important that the reader makes the transferability judgment, since their specific settings are unknown to us. The theory used is well recognised and research in this field supports our findings. An experienced psychiatric nurse who is the main author was central to the planning and implantation of the study. She also conducted the interviews. The researchers had considerable clinical experience in addition to research background. We believe this study can provide useful information on the well-being of adolescents who have a high use of non-prescription analgesics.

## Conclusion

Social exclusion and bullying have a negative effect on the participating adolescents’ thoughts and feelings. This might result in pain and reduced well-being, which can be considered mutually reinforcing reactions. From this perspective, pain might be one consequence of being socially excluded and bullied. Further, the use of non-prescription analgesics can then be a ‘tool’ used to treat the pain. Expressing pain might also provide an exit from stressful situations. Teachers and school nurses should pay attention to psychosocial stresses caused by social exclusion and bullying in relation to adolescents who have frequent pain and high use of non-prescription analgesics without a specific underlying disease or injury.

### Implications

School staff and school nurses should be aware of adolescents with frequent pain and high use of non-prescription analgesics. By mapping and identifying social exclusion and bullying, threats to adolescent’s well-being can be uncovered and targeted interventions can be implemented.

## Data Availability

The data that support the findings of this study are not available. Restrictions apply to the availability of these data, which were used under license for the current study, and so are not publicly available. The material is stored at Faculty of Health and Science, Oslo Metropolitan University until October 2020.

## Abbreviations

WHO: The World Health Organization
REC: The Norwegian Regional Committees for Medical and Health Research Ethics
